# Explainable artificial intelligence in orthopedic surgery

**DOI:** 10.1002/jeo2.12103

**Published:** 2024-07-17

**Authors:** Felix C. Oettl, Jacob F. Oeding, Kristian Samuelsson

**Affiliations:** ^1^ Department of Orthopaedic Surgery Hospital for Special Surgery New York New York USA; ^2^ Schulthess Klinik Zurich Switzerland; ^3^ School of Medicine Mayo Clinic Alix School of Medicine Rochester Minnesota USA; ^4^ Department of Orthopaedics, Institute of Clinical Science, The Sahlgrenska Academy University of Gothenburg Gothenburg Sweden

**Abbreviations**
AI: artificial intelligence
EBM: explainable boosting machine
GA2M: generalized additive models
ML: machine learning

Explainability refers to the ability of a machine learning (ML) model's decisions (and the process by which it makes those decisions) to be understood by humans; in this case, physicians and other healthcare workers. With the increasing deployment of artificial intelligence (AI) models in healthcare, the explainability and interpretability of these models have garnered increased scrutiny, and for good reason. This can be attributed to the high‐stakes environment that is the very nature of healthcare, with professionals making high‐impact decisions on a regular basis. The use of decision support systems and other automation with the help of AI has the potential to revolutionize the hospital landscape. However, a common problem when we look at such ML models is the lack of explainability, with the model receiving the data and returning an output, without any kind of explanation of how it got to said decision. This is what we call “black box” models. Concerns around this characteristic of AI center around the following three principles: [1] (1) Limited Trust and Adoption: Healthcare professionals, accustomed to transparent and justifiable decision‐making processes, may exhibit resistance toward adopting AI recommendations if the underlying reasoning remains opaque. This undermines trust and hinders the potential benefits of these technologies; (2) Exacerbation of Bias: Black box models are susceptible to perpetuating biases present in the training data, leading to discriminatory or unfair outcomes for specific patient groups. Without understanding the model's decision‐making process, identifying and mitigating such biases becomes exceedingly challenging; (3) Debugging Difficulties: When an AI model makes an erroneous prediction, pinpointing the root cause becomes a formidable task without insights into its internal workings. This hinders the ability to rectify errors and improve model performance.

The solution for these concerns comes in the form of explainable AI. Given the critical need for transparency and explainability in healthcare decisions, explainable AI is rapidly evolving. Techniques like saliency maps and explainable boosted machines are being actively explored to improve the interpretability of AI models [2, 3, 5]. Saliency maps are visual representations that highlight regions in an image or data point that significantly influence the model's decision, offering valuable insights into its focus areas. For instance, in medical imaging analysis, saliency maps can pinpoint specific regions in an X‐ray that the model identified as indicative of a particular pathology. Similarly, Shapley values provide one way to quantify the contribution of different input features to the final prediction of a model based on tabular data, like patient demographics, comorbidities, and other risk factors for developing a certain disease. Shapley values reveal the relative importance of potential predictive factors and can also depict potential nonlinear relationships between them. This nuanced understanding allows healthcare professionals to evaluate the model's reasoning and identify potential areas for improvement. Explainable boosting machines (EBMs) are categorized as generalized additive models (GA2M), making their decision‐making process more interpretable [4]. This allows healthcare professionals to trace the model's reasoning steps and understand how it arrives at its conclusions. That being said, achieving perfect explainability might not always be feasible, as it can sometimes come at the cost of sacrificing model accuracy. Striking a balance between these two competing objectives is an ongoing area of research.

Even explainable AI methods can be susceptible to perpetuating biases if the underlying design choices or training data are biased. Addressing this challenge requires careful consideration of ethical principles throughout the entire AI development process. For example, steps should be taken to ensure the prevention of model overfitting, and the model should be tested on an external set of data with differing characteristics such as geographic source, practice setting, or patient demographics.

Despite these challenges, explainable AI research is rapidly evolving, with promising advancements emerging. These include counterfactual explanations, which explore hypothetical scenarios where specific input features are altered and allow healthcare professionals to understand how the model's prediction would change. This can provide valuable insights into the model's reasoning and potential biases. Causal AI is another emerging field that aims to go beyond explaining correlations to understanding causal relationships between variables. By incorporating causal reasoning into explainable AI methods, we can gain a deeper understanding of how AI models arrive at their decisions and ensure they are aligned with established medical knowledge.

In conclusion, the responsible and ethical integration of AI in healthcare hinges on addressing the explainability challenge. By embracing explainable AI techniques and fostering a culture of transparency, we can harness the power of AI while safeguarding patient well‐being, building trust, and paving the way for a future of responsible and trustworthy AI‐supported healthcare. This journey requires continuous research, collaboration between diverse stakeholders, and a commitment to upholding ethical principles throughout the development and deployment of AI technologies in healthcare.

## AUTHOR CONTRIBUTIONS

All listed authors have contributed substantially to this work. Felix C. Oettl and Jacob F. Oeding performed the literature review and primary manuscript preparation. Editing and final manuscript preparation were performed by Kristian Samuelsson. All authors read and approved the final manuscript.
